# Functional Characterization of an *IL2RG* Variant, a Case Report of X‐Linked T‐ B + NK + SCID

**DOI:** 10.1002/iid3.70307

**Published:** 2025-12-29

**Authors:** Kristian Assing, Emil Birch Christensen, Christoffer Dellgren, Kerstin Kathrine Soelberg, Christina Fagerberg, Ida Coordt Elle, Bjørk Ditlev Larsen, Dorthe Grosen, Claire Booth, Hanne Vibeke Marquart, Tania Nicole Masmas, Hans Jakob Hartling

**Affiliations:** ^1^ Department of Clinical Immunology Odense University Hospital (OUH) Odense Denmark; ^2^ Department of Genetics Odense University Hospital (OUH) Odense Denmark; ^3^ Department of Pediatrics Odense University Hospital (OUH) Odense Denmark; ^4^ Infection, Immunity and Inflammation Department UCL Great Ormond Street Institute of Child Health, UCL London UK; ^5^ Department of Clinical Medicine, Faculty of Health and Medical Sciences University of Copenhagen Copenhagen Denmark; ^6^ Department of Clinical Immunology Tissue typing laboratory Copenhagen University Hospital Copenhagen Denmark; ^7^ The Child and Adolescent Clinic Copenhagen University Hospital Copenhagen Denmark

**Keywords:** B cells, case report, NK cells, pSTAT5, X‐SCID

## Abstract

**Objectives:**

Pathogenic variants in *IL2RG*, encoding the common γ chain (γ_c_/CD132), usually lead to T − B + NK‐ X‐SCID but can, occasionally, generate a T − B + NK+ phenotype. We wanted to delineate potential mechanisms for this discrepancy.

**Methods:**

The immunological work‐up of our patient comprised: whole genome sequencing and subsequent bioinformatics, flow‐cytometry (lymphocyte surface receptor expression, STAT5 phosphorylation, NK cell proliferation and degranulation), lymphocyte stimulation (IL‐2, IL‐4 and IL‐15) as well as restriction enzyme digestion and fragment size separation (evaluation of relative WT/variant expression).

**Findings:**

Our patient, hemizygous for a maternally derived, c.677 G > A *IL2RG* missense variant displayed a T − B + NK+ phenotype, no dysmorphic features and no thymus. The γ_c_ was surface expressed. In contrast to B cells from cord‐blood and adults (including maternal B cells), only pre‐gene therapy (patient) B cells did not decrease IL‐4Rα surface expression upon IL‐4 stimulation, consistent with compromised IL‐4R (and γ_c_) function. After gene therapy, patient B cells decreased IL‐4Rα upon Il‐4 stimulation. Pre‐gene therapy NK cells displayed normal, K562 cell, induced degranulation and, in response to IL‐2 and IL‐15 and exhibited normal initial pSTAT5 kinetics but clearly attenuated activation and proliferation day six. By restriction enzyme digestion and fragment size separation, selected T and B cells from the healthy mother exhibited skewed expression (92% and 84%, respectively) of the WT *IL2RG* allele.

**Conclusion:**

The selective WT *IL2RG* expression in maternal B cells was consistent with the compromised IL‐4R signaling (and compromised IL‐21R signaling) in her offspring (the patient), as both IL‐4 and IL‐21 are critical for normal human B cell germinal center reactions but not for peripheral B cell homeostasis. The c.677 G > A *IL2RG* variant permitted normal NK cell degranulation and initial STAT5 phosphorylation but was incapable of sustaining normal NK cell activation and proliferation in vitro. As IL‐2 and IL‐15 induced in‐vitro NK cell proliferation is primarily mediated through the low affinity βγ_c_ (CD122‐CD132) complex, our data indicate the importance of high affinity IL‐15Rα and IL‐2 Rα mediated signaling in‐vivo for sustaining NK cell numbers in X‐linked SCID. Consequently, we further hypothesize that in cases of X‐linked SCID, where even initial IL‐15 and IL‐2 STAT5 phosphorylation is compromised, in‐vivo trans‐presentation of IL‐15 (and IL‐2), via the high affinity IL‐15Rα and IL‐2Rα receptor subunits, will not be able to sustain normal peripheral NK cell numbers”.

## 1 Introduction

X‐linked severe combined immunodeficiency (X‐SCID) is caused by variants in the *IL2RG* gene, encoding the interleukin‐2 receptor gamma chain (CD132), also referred to as the common γ chain (γ_c_) [1]. As a subunit of various cytokine receptor complexes (IL‐2, IL‐4, IL‐7, IL‐9, IL‐15, and IL‐21), the γ_c_ plays critical roles in T and NK cell development and function [1]. Patients with loss‐of‐function (LOF) *IL2RG* variants are therefore at risk for lethal viral, bacterial and fungal infections and without hematopoietic stem cell transplantation [2] or gene therapy [3], this condition is usually lethal within the first 2 years of life [4].

The *IL2RG* gene consists of eight exons [5]. Approximately 25% of *IL2RG* gene variants are clustered in exon 5, which encodes a region of the extracellular domain important for cytokine binding mediated by the highly conserved WSXWS motif [6]. The WSXWS motif (Trp‐Ser‐Xaa‐Trp‐Ser) is also important for proper protein folding and intracellular transport of the γ_c_ protein [6]. In γ_c_ containing receptors, the γ_c_ associates with Janus kinase 3 (JAK3) whereas the IL‐2Rβ subunit (CD122, for IL‐2R and IL‐15R) or the specific cytokine receptor subunit (the remaining γ_c_ containing receptors) associates with JAK1 [1] [5]. Upon binding of cytokines to γ_c_ containing receptors, cross‐phosphorylation of JAKs is followed by phosphorylation of the IL‐2Rβ subunit/specific cytokine receptor subunit and the γ_c__[_ 1]. This leads to recruitment and phosphorylation of STAT proteins enabling their dimerization and subsequent translocation to the nucleus where they bind to regulatory elements and stimulate target genes transcription [5]. Variants in *IL2RG* predominantly lead to a T − B + NK‐ SCID phenotype [7], however *IL2RG* variants have been described with preserved peripheral NK cells [8, 9]. In this report, we describe a patient with an *IL2RG* variant in exon 5 with complete absence of T cells but with normal peripheral NK cell concentrations. To address this discrepancy, we performed flow‐cytometric cytokine receptor profiling and functional analysis of maternal and patients B and NK cells in conjunction with studies on relative allele (WT *vs. IL2RG* variant) expression in maternal T and B cells.

## 2 Methods

### 2.1 Next Generation Sequencing Technology

DNA was extracted from the peripheral blood samples with Tecan Freedom EVO® (Tecan, Switzerland). DNA libraries were quantified using the Qubit dsDNA BR assay Kit (Thermo Fisher Scientific, MA, USA). For WGS library preparation, we used Illumina DNA PCR‐Free Library Prep (Illumina, CA, USA). The prepared libraries were pooled and paired‐end sequenced on the NovaSeq. 6000 platform (Illumina, CA, USA) following the manufacturer's protocol. Sequencing data were mapped to the GRCh38 reference genome. The mean depth achieved by WGS was 60.8x. The WGS data were processed using the Illumina DRAGEN Bio‐IT platform (Illumina, CA, USA). Variant filtering was carried out using VarSeq. 2.3.0 (Golden Helix, MT, USA). Variants in the coding parts of the patient's genome were compared with parents'.

Variants that follow the expected patterns of inheritance were assessed. Variants that are reported as pathogenic/likely pathogenic in the ClinVar and HGMD databases and variants with PHRED‐scores ≥ 30 in disease‐associated genes according to the OMIM database and PanelApp were also assessed.

The whole genome was analyzed for copy number variants > 50 kb, and genes associated with known monogenic disease were analyzed for copy number variants > 1 kb.

### 2.2 Lymphocyte Marker Study

All antibodies (directly conjugated) were derived from Becton and Dickinson (New Jersey, USA). For flow‐cytometric evaluation of lymphocytes, the following antibodies were used: CD3 (FITC or PerCP‐Cy5‐5), CD4 (APC or PE‐Cy7), CD5 (PE‐Cy7), CD8 (APC or APC‐Cy7), CD19 (APC), CD16/CD56 (PE), CD27 (PE), CD31 (PE), CD45 (PerCP‐Cy5‐5), CD45RA (APC or FITC), CD45RO(PE), CD62L (PE), TCRαβ (FITC), TCRγδ (PE), HLA‐DR (APC), IgD (FITC), κ‐chain (FITC), λ‐chain (PE). For cytokine receptor surface expression, forward‐side scatter characteristics identified lymphocytes. Subsequently, NK cells were identified as CD3 (PerCP) and CD19 (APC) negative. The following cytokine receptor antibodies were used: CD122 (IL‐2Rβ, PE), CD124 (IL‐4Rα, PE), CD132 (γ_c_, PE), CD215 (IL‐15Rα, BV421) and CD360 (IL‐21R, PE). A Becton and Dickinson FACSCanto II Flow Cytometer (New Jersey, USA) was used.

### 2.3 Isolation of Maternal CD19 + B Cells and CD3 + T Cells Using RoboSep^TM^

EasySep HLA Chimerism Whole Blood CD3 Positive Selection Kit (StemCellTM, Vancouver, Canada) and EasySep HLA Chimerism Whole Blood CD19 Positive Selection Kit (StemCellTM, Vancouver, Canada) were used to isolate CD3+ or CD19+ cells respectively from EDTA stabilized full blood samples according to the manufacturer's protocol. According to the manufacturer´s specifications, isolation from whole blood yields CD3 + T cells of 99.1% ± 0.6 purity and CD19 + B cells of 98.6% ± 0.9 purity.

### 2.4 mRNA Extraction and cDNA Synthesis of Wild Type (WT) and Variant IL2RG Derived From Maternal B and T Cells

Extraction of mRNA from CD3+ , CD19+ as well as unseparated cells was performed using Maxwell RSC simplyRNA Blood kit (Promega, Madison, Wisconsin, USA) on a Maxwell^TM^ 16 system (Promega, Madison, Wisconsin, USA) according to the manufacturer's protocol. DNAse I (Promega, Madison, Wisconsin, USA) was added during mRNA extraction. First strand cDNA synthesis was done using Promega GoScript Reverse Transcription System (Promega, Madison, Wisconsin, USA) according to the manufacturer's protocol, on an Applied Biosystems Veriti Dx 96‐Well Thermocycler (Thermo Fisher Scientific, Waltham, Massachusetts, USA).

### 2.5 PCR, Restriction Enzyme Digestion and Fragment Size Separation of Wild Type (WT) and Variant *IL2RG* Derived From Maternal B and T Cells

A forward probe labeled with FAM fluorescence (FAM‐5'‐ ATAGACATAAGTTCTCCTTGC‐3') and a reverse primer targeting either mature mRNA (5'‐ACAGGAAAGGATTCTCTTTTG‐3') or pre‐mRNA (5'‐ CAAGTTTAGGGGCTTTAGTG‐3') was user in a PCR amplification of a fragment of the gene coding for IL2RG.

Following amplification, PCR products were digested with the restriction enzyme AciI (New England Biolab, Ipswich, Massachusetts, USA) according to the manufacturer's protocol. Target sequence for Acil is disrupted by the NM_000206.3: c.677 G > A variation, consequently only wild type sequences are cleaved [10].

Fragments were size separated on an ABI3500 genetic analyzer (Thermo Fisher Scientific, Waltham, Massachusetts, USA) detecting the FAM‐labeled probe, and peak area of cleaves and uncleaved fragments compared to calculate wild type to variant ratio per cell population.

### 2.6 NK Cell Degranulation

To assess the level of degranulation of NK cells, freshly isolated peripheral blood mononuclear cells (PBMC) (1 × 105 cells) were co‐cultured with K562 cells (1 × 105 cells) or phorbol 12‐myristate 13‐acetate (PMA)/Ionomycin cocktail in the presence of anti‐CD107a in U‐bottomed 96‐well plates in R10 media at 37°C. After 1 h, Monensin‐1 (BioLegend) was added, and the cells were incubated for an additional 2 h. The cells were then washed in FACS buffer (0.5% bovine serum albumin in PBS) and stained for CD3, CD14, CD19, CD56, CD16, CD57, NKG2A, CD69, and CD25 markers for 20 min at 4°C. Subsequently, the cells were washed in FACS buffer and analyzed by flow cytometry (ID7000, Sony Biotechnologies).

### 2.7 NK Cell Proliferation

To assess the proliferation of NK cells in response to cytokine stimulation, we used CellTrace labeling and dye dilution to trace individual generations of NK cells. First, freshly isolated PBMCs were labeled with 5 µM CellTrace Violet (Thermo Fisher Scientific) in PBS and incubated at 37°C for 20 min. The reaction was then quenched by adding R10 media and incubating at 37°C for additional 5 min. The cells were then washed and plated in U‐bottomed 96‐well plates (2 × 105 cells/well) and supplemented with indicated concentrations of IL‐2 or IL‐15. The cells received fresh R10 media and cytokine supplements at day 3. On day 6, the cells were washed in FACS buffer and stained for CD3, CD14, CD19, CD56, and CD16 markers for 20 min at 4°C. Afterward, the cells were washed in FACS buffer and analyzed by flow cytometry (ID7000, Sony Biotechnologies). CD3‐ CD14‐ CD19‐ CD16 + /CD56 + NK cell proliferation was modelled and analyzed using FlowJo software (BD Biosciences).

### 2.8 IL‐2 and IL‐15 Induced STAT5 Phosphorylation

To assess the phosphorylation of STAT5 in response to stimulation of the IL‐2 and IL‐15, thawed viable PBMCs (1 × 10^6^ cell/mL) were stimulated for 15 min with IL‐2 (30 or 300 ng/mL), IL‐15 (5 or 50 ng/mL) or were left unstimulated at 37°C in a 5% CO2 incubator in FACS tubes. Subsequently, the PBMCs were fixed using Fix Buffer (Becton and Dickinson) followed by staining for surface markers (CD3, CD4, CD56,) for 15 min at room temperature, washed in FACS Flow (including BSA) and suspended in Perm Buffer IV (Becton and Dickinson) for 20 min at room temperature.

Fixated and permeabilized PBMCs were washed two times in FACS Flow (including BSA) and incubated with pSTAT5 antibody (Becton and Dickinson) for 30 min followed by a wash in FACS Flow (including BSA) and resuspension in FACS Flow and finally analyzed by flow cytometry (Navios).

### 2.9 Interleukin‐4 Stimulation of Patient, Adult and Cord‐Blood CD19 + B Cells

PBMC (1 × 106 cell/mL) were left unstimulated or stimulated with IL‐4 (Becton and Dickinson, 100 U/mL in RPMI medium with (5%) AB‐serum) for 3 h in 96 well plates at 37°C in a 5% CO2 incubator. Subsequently, surface IL‐4R expression between unstimulated and IL‐4 stimulated CD19 + B cells for patient, adult and cord‐blood CD19 + B cells was determined flow‐cytometrically.

### 2.10 Results Format

Values for surface cytokine receptor expression are given as median fluorescence intensity (MFI) values and grouped values are presented as means and standard deviations.

### 2.11 Ethical Permissions

With respect to publication, written informed consent was obtained from the proband's parents. Publishing was permitted by the chairman of the Regional Committee on Health Research Ethics for Southern Denmark (S‐20192000‐48).

## 3 Results

### 3.1 Initial Immune Status of the Proband and Mother

The proband (male infant) was born at term and identified through the Danish national newborn SCID screening program where PCR analysis of signal joint T cell Receptor Excision Circles (sjTRECS) performed on a dried blood spot sample revealed reduced sjTRECS concentrations of 30 copies/10^5^cells (cut‐off for SCID is ≤ 50 copies/10^5^cells) [11]. A subsequent flow‐based lymphocyte marker study showed absence of peripheral CD3 + CD4 + CD45RA + CD31+ recent thymic emigrants (RTE) and CD3 + CD4 + /CD8 + T cells but normal CD19 + B cells (0.6 ×109/L) and CD3‐ CD16 + /CD56 + NK cell concentrations (0.7 × 109/L). Peripheral B cells showed a normal surface kappa (56% of CD19 + )/lambda (42% of CD19 + ) distribution and normal differentiation for age as evidenced by predominance of CD19 + CD5 + CD27‐ B cells (76% of CD19 + ) and CD19+ IgD+ CD27‐ (naïve) B cells (98% of CD19 + ). The patient had no dysmorphic features. Imaging diagnosticss did not visualize a thymus. The patient was home‐isolated when identified though the newborn screening program (based on the dried blood spot samples) and started antimicrobial prophylaxis with trimethoprim/sulfamethoxazole against pneumocystis pneumonia, acyclovir against and Herpesviridae, and antifungal treatment with fluconazole or itraconazole. He, furthermore, started immunoglobulin replacement treatment despite normal immunoglobulins 1 month prior to gene therapy in accordance with the gene therapy protocol. He was infected with Rhinovirus 1‐month‐old; clinically he only revealed mild upper respiratory infection symptoms. He was only tested Rhinovirus negative on nasopharyngeal after gene therapy. He experienced no other infections and no autoimmunity. When the patient was 6‐month‐old, he received lentiviral vector based gene therapy after non‐myeloablative conditioning with intravenous busulfan at Great Ormond Street, London, UK. There were no complications or infections in relation to or after gene therapy. Currently, now 1½ year after gene therapy, he is thriving, developing according to age, and has started in nursery. His peripheral CD4+ and CD8 + T cell compartments are reconstituted (data not shown).

Lymphocytes analysis from the patient's healthy mother (carrier) displayed normal concentrations of CD4 + T cells, CD8 + T cells, CD19 + B cells and CD3‐ CD16 + /CD56 + NK cells as well as normal subset (naïve *vs.* memory) distributions of CD4 + , CD8 + T cells and CD19 + B cells (data not shown).

### 3.2 Genetic Work‐up

**Figure 1:**
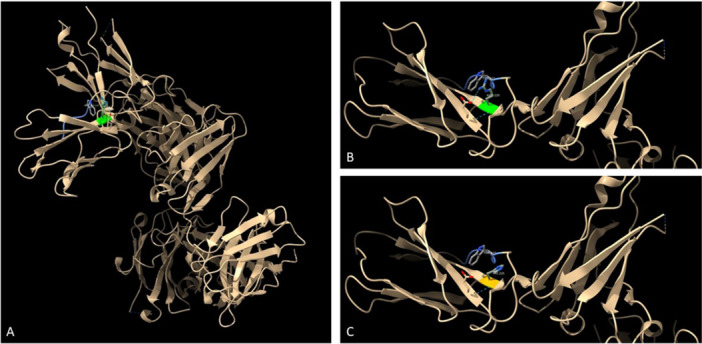
Structural analysis of the IL2RG protein using ChimeraX. Model PDB_9JQT (based on electron microscopy at 2.7 Å resolution). Structure editing using Rotamer function with most relaxed Chi score (highest prevalence). (A) Overview of the wildtype protein. (B) Zoom of the affected domain of the wildtype protein including the WSXWS motif. (C) Predicted structure with the p. Arg226His variant including the WSXWS motif. Green: Wildtype Arg226, Yellow: Mutant His226, Light blue: WSXWS motif, Turquoise dashed lines: Predicted H‐bonds, Grey: Amino acid side chains of the WSXWS motif and affected amino acid position, Red: Oxygen atoms, Blue: Nitrogen atoms.

Whole genome sequencing revealed the patient to be hemizygous for a maternally derived missense variant (c677G > A; p. Arg 226 His) in exon 5 of the *IL2RG* gene. The variant had a CADD‐score of 26.8 and four (SIFT, PolyPhen 2, MutationTaster and FATHMM) out of five prediction algorithms predicted the variant to impair protein function. The variant has previously been described as pathogenic (American College of Medical Genetics and Genomics (ACMG) classification: C5, reference) and as affecting cytokine binding due to its vicinity to the WSXWS motif encoded by exon 5 [10]. We have modelled the 3D structure (using ChimeraX) of the wild type and variant IL2RG protein, highlighting the position of the Arg/His 226 residue relative to the WSXWS motif (Figure 1).

### 3.3 Cytokine Receptor Profiling of Patient, Adult and Cord‐Blood Lymphocytes

**Figure 2:**
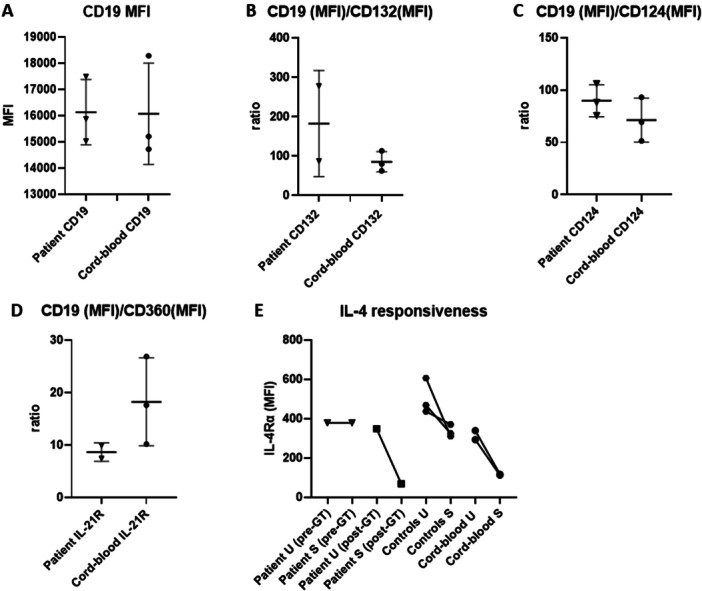
B cell surface expression of γ_c_ interacting receptor subunits and IL‐4 responsiveness Dot plots showing expression (median fluorescence intensity (MFI); mean ± SD) of γ_c_ interacting receptor subunits on patient B cells as compared to cord blood and adult B cells. Pre‐gene therapy (GT) patient and cord blood B cells surface expressed CD19 equally (A) hence surface expression of γ_c_ (CD132, (B), IL‐4Rα (CD124, (C) and IL‐21R (CD360, (D) were expressed relative to CD19 surface expression as ratios. (E) Only pre‐GT patient B cell were non‐responsive to 3 h of IL‐4 stimulation (100 U/mL) and did not downregulate IL‐4Rα. U = unstimulated. S = stimulated.

Patient (pre‐gene therapy (GT)) and cord‐ blood B cells expressed similar levels of surface CD19 (Figure 2A). Hence, surface expression of cytokine receptor proteins was calculated relative to the CD19 surface expression (CD19 (MFI)/cytokine receptor protein (MFI) ratios: mean; SD). Pre‐GT and cord‐blood B cells did not segregate with respect to γ_c_ (CD19/CD132 ratio: Figure 2B) and IL‐4Rα (CD19/IL‐4Rα ratio: Figure 2C) surface expression. Pre‐GT B tended towards increased IL‐21R surface expression compared to cord‐blood B‐cells (CD19/IL‐21R ratio: Figure 2D). In contrast to adult (including the proband's mother and father) and cord‐blood B cells, pre‐GT B cells did not display IL‐4 responsiveness, as their IL‐4Rα expression was not decreased in response to 3 h of IL‐4 stimulation (Figure 2F). One year post‐GT however, B cells displayed pronounced downregulation of their IL‐4Rα upon IL‐4 stimulation (Figure 2E).

### 3.4 NK‐Cell Proliferation, Cytotoxicity and Differentiation

**Figure 3:**
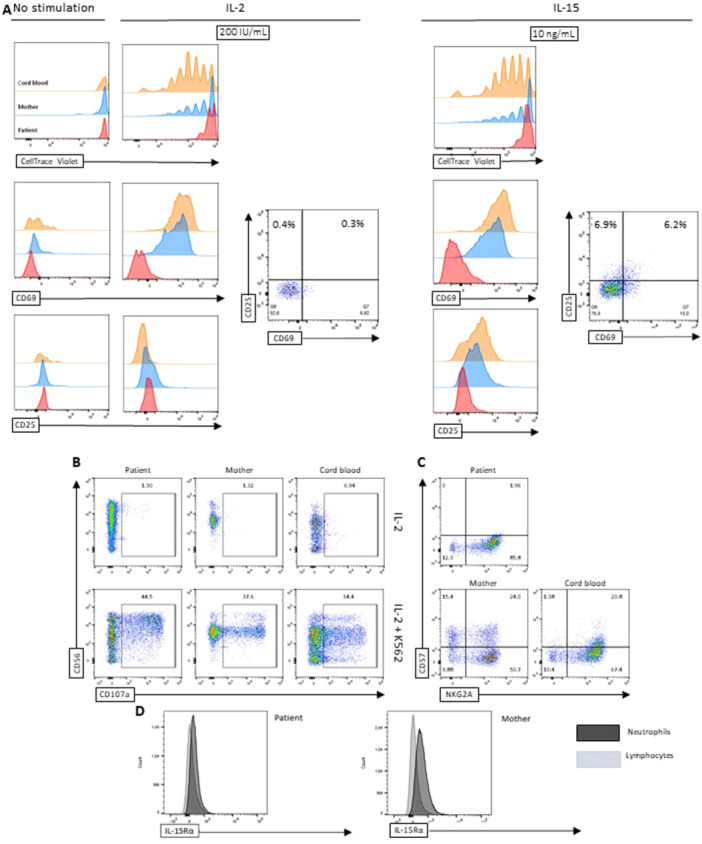
Patient pre‐GT NK cells were unresponsive to IL‐2 and IL‐15 stimulation but maintained natural cytotoxicity. (A) Purified PBMC's from patient (pre‐GT) and healthy control samples were labelled with CellTrace Violet and cultured in the presence of IL‐2 (20‐200 IU/mL, only 200 IU/mL is shown), IL‐15 (1‐10 ng/mL, only 10 ng/mL is shown), or no added cytokines. NK cell proliferation was assessed by flow cytometry (histograms) on day 6. NK cells were simultaneously examined for surface expression of the activation markers CD25 and CD69 (histograms and dot‐plots). CD25 and CD69 surface expression was relatively sparse (histograms) but measurable on patient NK cells (dot‐plots) in response to 6 days of IL‐15 stimulation. (B) Purified PBMC's were co‐cultured with K562 cells at a 1:2 ratio in the presence of IL‐2 (100 IU/mL) or IL‐2 alone for 3 h. NK cell degranulation was then examined by surface expression of CD107a on gated NK cells. (C) NK cells were analyzed for surface expression of maturation markers NKG2A and CD57, to mark the presence of immature and terminally differentiated NK cell subsets respectively. (D) Histograms depicting surface IL‐15Rα expression on peripheral blood lymphocytes and neutrophils from the patient and the patient´s mother.

In comparison with adult (we used the patient's healthy mother as control as her NK‐cell immune‐profile was representative of other adult NK‐cells, data not shown) and cord blood NK cells, pre‐GT NK cells responded with markedly less proliferation, and upregulation of the activation markers CD25 and CD69, to IL‐2 (20 IU/mL and 200 IU/mL) and IL‐15 (1 ng/mL and 10 ng/mL) stimulation (Figure 3A, histograms), but not to stimulation with PMA and ionomycin (data not shown). However, pre‐GT NK cells displayed measurable CD25 and CD69 surface expression upon IL‐15 stimulation but not IL‐2 stimulation (Figure 3A, dot‐plots), thereby mimicking the activation pattern displayed by control NK‐cells (not shown). Pre‐GT NK cells displayed pronounced degranulation (CD107a expression) in response to K562 cells, both in the presence and absence of IL‐2 (Figure 3B only showing in the presence of IL‐2). Pre‐GT and cord‐blood CD3‐ CD56 + NK cells displayed similar low frequencies of NKG2A + CD57+ double positive NK cells, thus being clearly differentiable from the higher frequencies of NKG2A+ and CD57+ double positive NK cells among adults (Figure 3C). Human neutrophils constitutively express IL‐15Rα [12]. In contrast, human peripheral lymphocytes, apart from CD8 memory subsets, do not consistently display IL‐15Rα expression ex‐vivo [13] [14]. As visualized by Figure 3D, both the patient´s and the mother´s neutrophils displayed, when compared to their respective lymphocytes, a right‐shift in BV421 intensity consistent with neutrophil surface expressed IL‐15Rα.

### 3.5 NK Cell STAT5 Phosphorylation in Response to IL‐2 and IL‐15

**Figure 4:**
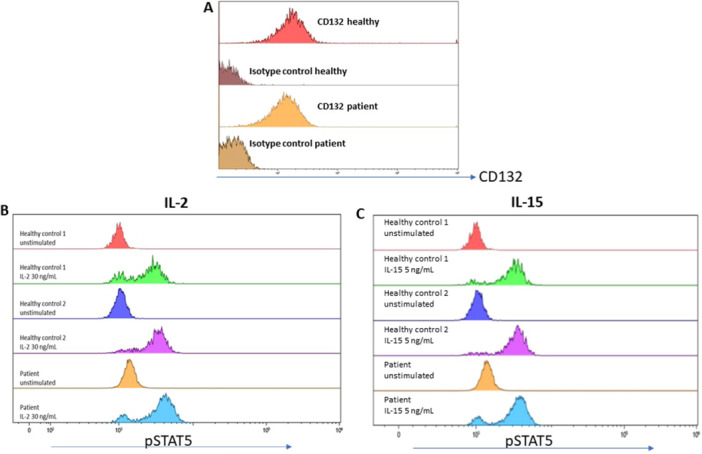
Pre‐GT NK cell γ_c_ surface expression and STAT5 phosphorylation in response to IL‐2 and IL‐15 Patient (pre‐GT) CD3‐ CD56 + NK cells displayed comparable γ_c_ (CD132) surface expression to adult controls NK cells (A). In response to 15 min of stimulation with (B) IL‐2 (30 ng/mL) and (C) IL‐15 (5 ng/mL), patient NK cells demonstrated the same extent of STAT5 phosphorylation as CD3‐ CD56 + NK cells from two adult controls.

In response to 15 min of stimulation with IL‐2 (30 ng/mL and 300 ng/mL, respectively) and IL‐15 (5 ng/mL and 50 ng/mL respectively), pre‐GT CD3‐ CD56 + NK cells, (displaying similar γ_c_ surface expression to adult controls NK cells, Figure 4A), demonstrated comparable STAT5 phosphorylation to CD3‐ CD56 + NK cells from two adult controls (Figure 4B, shown are only the results for the low IL‐2 and IL‐15 concentrations).

### 3.6 Frequency Distribution of WT and Variant *IL2RG* cDNA in Maternal CD19 + B‐Cells and CD3 + T‐Cells

**Figure 5:**
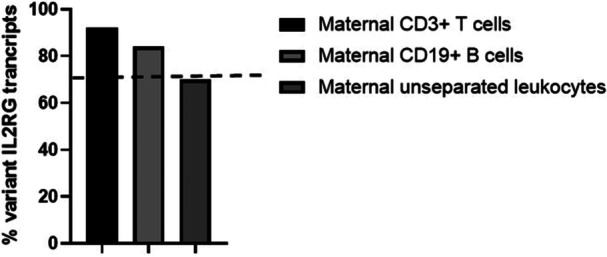
Distribution of variant *IL2RG* expression among peripheral maternal leukocytes. Distribution of variant IL2RG cDNA among IL2RG cDNA transcripts derived from peripheral maternal CD3 + T cells, maternal CD19 + B cells as well as unseparated peripheral maternal leukocytes. The dotted line represents the 75% limit separating skewed from non‐skewed expression.

The recognition site of the restriction enzyme Acil: 5´C^**˅**^CGC 3´ is abolished in the c677G > A *IL2RG* variant, hence only WT *IL2RG* cDNA is digested by Acil. During female embryogenesis, skewed inactivation of the X‐chromosome occurs when more than 75% of somatic cells chose to express the X chromosome from the same parent [15]. However, female lymphocytes display a propensity for increased reactivation of inactivated chromosomes leading to increased bi‐allelic expression of immune related genes in particular [16]. Among isolated maternal CD3 + T cells, 92% of *IL2RG* mRNA transcripts were WT whereas among isolated maternal CD19 + B cells, 84% of the *IL2RG* mRNA transcripts were WT, hence expression of the *IL2RG* was markedly skewed in both lymphocyte subsets (Figure 5). Among the mother's unseparated whole blood leukocytes, skewed *IL2RG* expression was not observed, as the fraction of WT *IL2RG* among *IL2RG* transcripts was < 75% (70%, Figure 5).

## 4 Discussion

This is the first report of cytokine receptor and functional profiling of B cells respectively NK cells derived from an infant hemizygous for the previously reported c677G > A *IL2RG* missense variant *[* 5]. Similar to the case reported by Notarangelo et al [5], our patient also had preserved NK cell numbers and detectable surface expression of the γ_c_. The c677G > A *IL2RG* variant rendered patient NK cells highly capable of K562 induced degranulation but with markedly impaired activation and proliferation in response to IL‐2 and IL‐15. Enforced expression of Bcl‐2 rescued NK cell numbers in IL‐2Rβ‐deficient mice but failed to restore their NK cell cytotoxicity demonstrating IL‐2Rβ as being critical for NK‐cell cytoxicity [17]. Preserved K562 induced NK cell degranulation was therefore consistent with some functional capacity of our patient's IL‐2Rβ‐γ_c_ (CD122‐CD132) complex. In response to brief stimulation with different IL‐2 and IL‐15 concentrations, patient NK cells also displayed normal pSTAT5 kinetics. In the absence of the high affinity receptor subunits IL‐2Rα (CD25) or IL‐15Rα (CD215), IL‐2 and IL‐15 can bind the low affinity IL‐2Rβ‐γ_c_ complex with a K_d_ ≈ 1 nM [1]. Our lowest IL‐2 (30 ng/mL) and IL‐15 (5 ng/mL) concentrations, used for NK cell STAT5 phosphorylation, exceeded this K_d_ by far. However, the IL‐2 stimulation time used to access STAT5 phosphorylation was too short to enable IL‐2Rα (CD25) upregulation on human NK cells [18]. Soluble IL‐15 is barely detectable in humans and IL‐15 mediates its effects by binding irreversibly to the high affinity 15Rα receptor intra‐cellularly followed by trans‐presentation of IL‐15, via membrane bound 15Rα, to IL‐2Rβ‐γ_c_ expressing lymphocytes [19]. Membrane IL‐15Rα receptors are therefore not accessible for soluble IL‐15. Therefore, STAT5 phosphorylation was generated primarily through the low affinity IL‐2Rβ‐γ_c_ complex. As IL‐2Rβ is critical for NK cell development [19], preserved initial pSTAT5 kinetics could be critical for our patient's ability to sustain normal peripheral NK cell concentrations. After 6 days of IL‐2 stimulation, we observed very scant CD25 surface expression on proliferating NK cells in general. Therefore, the prolonged IL‐2 and IL‐15 stimulations (10 ng/mL of IL‐15 exceeding the 5 ng/mL used for pSTAT5 assessment) also primarily targeted the NK cell IL‐2Rβ‐γ_c_ complex, clearly showing a deleterious effect of the c677G > A *IL2RG* missense variant on IL‐2 and IL‐15 induced NK cell proliferation. Diminished NK cell proliferation could be due to several unexamined factors. We did not evaluate whether 1) STAT3 phosphorylation or STAT dimerization was compromised and 2) potential opposing effects of the suppressor of cytokine signaling (SOCS) system was not assessed. However, given the critical role of IL‐15 in human NK cell homeostasis [19], the sparse in‐vitro NK cell proliferation, conferred by IL‐15 supplementation, point to the importance of in vivo IL‐15Rα mediated IL‐15 trans‐presentation in sustaining our patient's normal peripheral NK cell concentrations. By protecting IL‐15 from degradation, IL‐15Rα markedly potentiates the biological activity of IL‐15 [20]. Consequently, we further hypothesize that in cases of X‐linked SCID, where even initial IL‐15 and IL‐2 STAT5 phosphorylation is compromised, in‐vivo trans‐presentation of IL‐15 (and IL‐2), via the high affinity IL‐15Rα and IL‐2Rα receptor subunits, will not be able to sustain normal peripheral NK cell numbers. Our finding that patient neutrophils expressed IL‐15Rα renders it plausible that intra‐nodal trans‐presentation of IL‐15, by neutrophil or dendritic cell surface expressed IL‐15Rα, could contribute to the homeostatic maintenance of NK cell numbers in the patient.

The patient's NK cells also responded with sparse though ready detectable upregulation of CD25 in response to IL‐15 suggesting that trans‐presentation of IL‐2 by CD25 in secondary lymphoid organs also could contribute to his NK cell homeostasis.

Interleukin‐7R signaling is critical for thymopoiesis [21]. The total absence of peripheral T cells in the patient indicated a detrimental effect of the Arg 226 His missense γ_c_ variant on IL‐7R signaling consistent with his mother's T cells predominantly making use of the WT γ_c_ allele. In our diagnostic setup, patient B cells displayed no signs of aberrant differentiation as evidenced by normal kappa and lambda light chain surface expression as well as an expected (for age) dominance of CD5^+^ B cells and a relative scarcity of CD27+ memory B cells. These findings are consistent with the lack of any irreplaceable role for γ_c_ cytokines in human B cell genesis [22] and with the central role for non‐ γ_c_ cytokines such as APRIL and BAFF in sustaining peripheral B cell numbers in humans [23]. In contrast to post‐GT B cells as well as adult and cord‐blood B cells, pre‐GT B cells were incapable of downregulating the IL‐4Rα upon stimulation with high concentrations of IL‐4, an observation consistent with compromised IL‐4Rα signaling in pre‐GT patient B cells. The skewed use of WT *IL2RG* in maternal B cells most likely reflect selection pressure and was consistent with the affected IL‐4R signaling, but potentially also with compromised IL‐21R signaling, in her offspring, as both IL‐4 and IL‐21 are crucial regulators of germinal centered B cell functions [24, 25]. X‐chromosome inactivation in females is thought to occur prior to the differentiation into a myeloid and lymphoid lineage at the stage when about eight embryonic founder cells are present [26]. The marked discrepancy in the frequency distribution of variant *IL2RG* cDNA between maternal B‐ and T‐cells versus maternal neutrophils (constituting the majority of cells among unseparated maternal whole blood leukocytes), suggests that this discrepancy was due to additional factors than skewed X‐chromosome inactivation per se. This difference was likely functionally related, as maternal B and T cells, expressing the WT *IL2RG*, would have proliferative advantages over their variant *IL2RG* expressing counterparts. Hence, the dominance of WT *IL2RG* expressing maternal lymphocytes in the peripheral lymphocyte pool. The selective use of WT *IL2RG* in maternal B cells likely contradicts the findings by Deal et al [27], who described an X‐linked SCID patient, with a different γ_c_ variant (c.675 C > A, p. Ser225Arg, the variant being reported as likely pathogenic) than ours, but where all B cells were host derived and with functioning IL‐21 receptors, even 47 years post‐transplantation. If IL‐21R function is indeed compromised in pre‐GT B cells, it requires that our patient achieves a certain degree of B cell chimerism with respect to the WT *IL2RG* in order to ensure prober, T cell dependent, B cell function. Hopefully, this might already have occurred, as evidenced by the IL‐4 responsiveness regained by post‐GT B cells. There a several limitations to this study, we did not test whether IL‐15 induced STAT3 phosphorylation or STAT dimerization was affected in patient NK cells or whether IL‐21R signaling, by assessing STAT3 phosphorylation, was compromised in patient B cells. This was also partly due to very limited amounts of patient PBMC available. It will also be difficult to test our hypothesis regarding the importance of STAT5 phosphorylation for normal NK cell counts, as the low to absent NK cell numbers, in classical X‐linked SCID, makes testing this hypothesis difficult. As a case report, the findings reported herein might also be difficult to generalize. However, we think that the STAT5 and NK cell proliferation data, derived from this *IL2RG* variant case report, raise some interesting questions as to why NK cells might be preserved in this condition.

In conclusion, this case of X‐linked T‐B + NK + SCID, due to the c677G>A *IL2RG* missense variant, demonstrated attenuated IL‐4R signaling consistent with compromised B cell function as maternal B cells preferentially used the WT *IL2RG* allele and displayed an IL‐4 responsive IL‐4Rα. We have documented that this *IL2RG* variant preserves NK cell degranulation and permits normal initial STAT5 phosphorylation but is not capable of sustaining normal NK cell activation and proliferation in‐vitro. The latter indicates the central role for especially IL‐15Rα mediated trans‐signaling in‐vivo for sustaining peripheral NK cell numbers in X‐linked SCID, a process which likely also requires preserved STAT5 phosphorylation in response to IL‐15.

## Author Contributions

K.A. conceived of the case, wrote the major part of primary manuscript and devised experiments. E.C. performed functional NK analyses. C.D. performed variant and WT gene expression experiments on isolated maternal B and T cells. K.S. wrote (with K.A.) parts of the initial manuscript, C.F., I.C.E., and B.D.L. were responsible for whole genome analyses of patient and parents and 3D modelling. D.G. was responsible for patient care at Odense University Hospital. C.B. coordinated gene therapy and follow up at Great Ormond Street Hospital. T.M. was responsible for patient care and, in cooperation with Great Ormond Street, organized gene therapy. H.J.H. and H.M. performed the extensive STAT5 phosphorylation studies. All authors have read and agreed to the final manuscript.

## Conflicts of Interest

The authors declare no conflicts of interest.

## Data Availability

All data related to this study will be shared at the request of other investigators for purposes of replicating procedures and results, according to national and international GDPR rules and following individual DTA and MTA rules with relevant investigators.
